# Sonographic Diagnosis of Arterioportal Fistula

**DOI:** 10.1155/2010/430219

**Published:** 2011-01-18

**Authors:** Canan Alkim, Huseyin Alkim, Gonul Gurkaynak, Gulay Temucin

**Affiliations:** ^1^Department of Gastroenterology, Sisli Etfal Training and Research Hospital, 34360 Istanbul, Turkey; ^2^Department of Gastroenterology, Bakırkoy Dr. Sadi Konuk Training and Research Hospital, 34147 Istanbul, Turkey; ^3^Department of Gastroenterology, Turkey High Specialty Hospital, 06420 Ankara, Turkey

## Abstract

*Aim*. We aimed to identify and describe characteristic and diagnostic ultrasonographic features of arterioportal fistula cases. *Patients*. In this case series we describe 3 patients with arterioportal fistula. By depending on shared sonographic features of these patients we describe a “sonographic pattern” for the sonographic diagnosis of arterioportal fistula. *Conclusion*. In summary; both of the artery and vein related with fistula were wider than normal and seen as adjacent anechoic circles, there was an aneurismatic dilation on vein which has turbulent flow within it, the communication between the artery and aneurism can be seen sonographically, both of the vessels have arterial flow, filling of the vein was retrograde and other branches of the artery and vein unrelated with aneurism were all normal in dimension.

## 1. Introduction

Arterioportal fistula (APF) is a rare vascular disorder of the mesenteric circulation which is an abnormal direct communication between the mesenteric arteries and portal venous system [1]. Patients with APF may be asymptomatic or may present with a protean clinical syndrome including the complications of portal hypertension (gastrointestinal bleeding and ascites), heart failure, and intestinal ischemia [2]. APFs can be congenital or acquired. Congenital causes are very rare and associated with hereditary telangiectatic diseases (Osler-Weber-Rendu syndrome, Ehler-Danlos syndrome), biliary atresia, arteriovenous malformations and cavernous hemangiomas. Acquired causes of APF are blunt or penetrating trauma, iatrogenic procedures (like percutaneous liver biopsy, cholangiography, splenoportography, gastric and hepatobiliary surgery), hepatocellular carcinomas, cirrhosis and vasculitis syndromes, for example, Behçet's disease [1, 2]. Small APFs with low flow are generally an incidental finding, particularly in cases related with iatrogenic traumas, hepatocellular carcinomas and cirrhosis. On the other hand, large high flow APFs may cause serious portal hypertension and are one of the rare treatable causes of portal hypertension. For this reason early diagnosis of APFs, either asymptomatic or not, is very important [1–4]. 

 APF can be demonstrated by many imaging methods (ultrasonography, computerized tomography, magnetic resonance imaging), but accurate definitive diagnosis is made by arteriography. Ultrasonography (US) is a rapid, reliable and noninvasive modality for identifying APFs. In the literature, while presenting APF cases, some of the sonographic features were demonstrated [1–3]. Also, when presenting our first case, we summarized sonographic characteristics [5]. Then, we had a chance to observe another two cases, and we noticed that our three cases were shared certain sonographic features. In this paper, we aimed to identify and reemphasize on specific and diagnostic features of ultrasonography in APF cases.

## 2. Patients

**Patient 1**

A twelve-year-old girl was admitted with hematemesis and melena. This was her first bleeding episode. She did not have any symptom before. Also cardiac failure was not present. There was no history of abdominal trauma, hepatic disease or liver biopsy. Emergency upper endoscopy showed bleeding varices in the distal esophagus. At ultrasonography an aneurismatic dilation was detected within the left lobe of the liver. This aneurismatic dilation was on the left branch of the portal vein which was dilated also. The right branch of the portal vein, hepatic veins, and vena cava inferior were all normal. The common hepatic artery was dilated starting from the celiac trunk, and this dilation was continued on the left branch of the hepatic artery and ends in the aneurismatic dilation. Doppler US showed turbulent flow within the aneurism and arterial and retrograde flow in the portal vein. With all of these sonographic clues, diagnosis of APF was made. Angiography confirmed APF between the left branch of the hepatic artery and left portal vein. All of the other branches of the celiac truncus and superior and inferior mesenteric arteries were all normal angiographically. The patient was cured after surgical hepatic artery ligation. For detailed case story and US photographs can be looked to the previously published case report [5].

**Patient 2**

A 39-year-old male admitted to our hospital with swelling of abdomen and legs, hematemesis, melena, and weakness. This was his fourth episode of upper gastrointestinal bleeding. All of the attacks occurred in the last 1 year. He was treated before at another hospital with the diagnosis of esophageal variceal bleeding. This patient had a penetrating abdominal trauma 11 years ago which was treated with a liver repair operation.

On his physical examination, he was pale and weak. There were a palpable liver 4-5 cm below the right costal margin and splenomegaly 1-2 cm at the left costal margin. Also, we detected ascites at umbilicus level. Pretibial oedema was present at the legs. His cardiac examination was normal.

His initial laboratory testing results were as follows: hemoglobin: 3.8 gr/dl, hematocrit: 11.5%, white blood cells: 7200, platelets: 126.000, AST: 50 U/L, ALT: 15 U/L, total protein: 6.6 gr/dl, albumin: 2.7 gr/dl, cholesterol: 98 mg/dl and prothrombin time: 15 sec (55%). Hepatitis markers were all negative.

**Figure 1:**
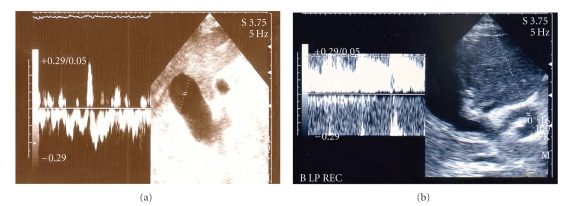
Ultrasonographic images of patient 2 showing (a) two adjacent anechoic circles within the right lob of the liver. The smaller one was dilated right hepatic artery, and the big one was the aneurismatic dilation on the right main branch of portal vein which has turbulent flow within it (b) The dilated and tortuous right hepatic artery ending in the aneurism.

 Esophageal variceal bleeding was seen at the upper gastrointestinal endoscopic examination, and band ligation was performed. Also gastric (fundal) varices were observed. At US, liver was larger than normal with increased echo pattern. The left branch of the portal vein was thrombosed; right branch dilated and formed an aneurism within the right lobe of the liver together with ascites and splenomegaly. Right hepatic artery was dilated all along its route, and this dilation was continued on common hepatic artery just starting at branching of the artery from the celiac truncus. Dilated artery was laying parallel with dilated portal vein. Also, we detected a fistula between the artery and the vein sonographically (Figure 1). In Doppler US, there was a turbulent flow within the aneurism, and arterial flow was demonstrated in both of the artery and the vein. The flow of portal vein was retrograde. The hepatic veins and vena cava inferior were found normal.

**Figure 2:**
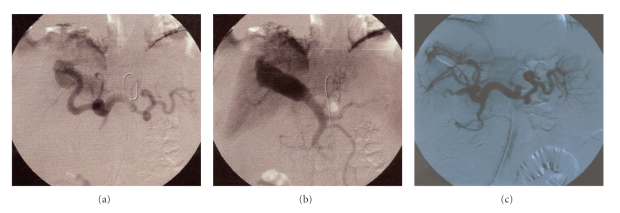
Angiographic images of patient 2 showing (a) dilated main hepatic artery and dilated right branch of the hepatic artery which ends in the aneurismatic dilation. The other branches of the celiac truncus and left branch of the hepatic artery were all normal in diameter (b) Aneurismatic dilation on the dilated right branch of the portal vein with retrograde filling. The other branches of portal system were all normal in diameter. (c) Control angiography of the patient, done 8 months after the detachable balloon treatment, showing complete occlusion of the arterioportal fistula.

Celiac angiography showed that the right branch of the hepatic artery and common hepatic artery were dilated all along their route just originating from the celiac truncus. A high flow fistula was demonstrated between the right hepatic artery and right portal vein at the aneurismatic region. Also there was a communication between the terminal branches of superior mesenteric artery and right hepatic artery. Portal venous system has retrograde filling at the arterial phase of angiography (Figures 2(a) and 2(b)). Splenic vein was dilated and tortuous. Spontaneous splenorenal shunt was present. Also, coronary vein was patent and there were a lot of collaterals at perigastric, perisplenic and distal esophageal region. Diameter of the superior mesenteric vein was also increased together with presence of some varicose veins at its branches. Because of his diminished liver function, presence of portal vein thrombosis, and many extrahepatic collaterals in the abdomen angiographic treatment was preferred. While deciding the type of angiographic intervention, detachable balloon application was preferred instead of coil embolization because of the risk of coil migration. Transarterial detachable balloon was successfully used to occlude high flow APF. Then, his esophageal and gastric varices were lost at the endoscopy. But, the patient readmitted to hospital eight months later with esophageal variceal rebleeding. Doppler US demonstrated that the fistula was closed with no flow within the aneurism. On control angiography (Figure 2(c)), it was seen that the angiographic treatment was successful. There was no flow at the aneurism region and the portal vein was not filled during celiac dye injection. So it was thought that his portal hypertension recurred because of existing portal vein thrombosis and possible liver damage due to long-term APF. The patient was followed up with conservative therapy.

**Patient 3**

A 59-year old multiparous female patient was admitted with nonspecific abdominal pain, nausea, vomiting and diarrhea for the last two or three months. No operation or trauma history was present. Her blood pressure was 90/60 mmHg, and pulse rate was 100/min. No abnormal finding was detected at cardiac and pulmonary examinations. Her abdomen was sensitive on palpation. Liver was not palpable, but spleen was palpable 3 cm at the left costal margin. Also, a systolic murmur was detected on the spleen and at the left back.

Laboratory tests were as follows: hemoglobin: 9.8 gr/dl, platelets: 283.000/L, white blood cells: 8800/L, AST: 47 U/L, ALT: 44 U/L, ALP: 523 U/L, urea: 287 mg/dl, creatinine: 1.1 mg/dl, total protein: 9.2 gr/dl, albumin: 4.6 gr/dl, total bilirubin: 1.2 mg/dl, and direct bilirubin: 0.5 mg/dl. 

**Figure 3:**
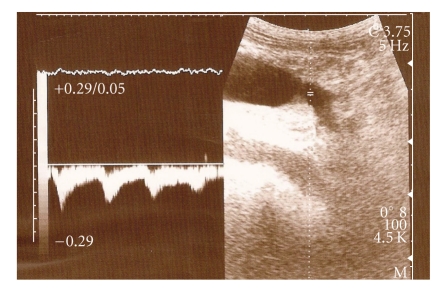
Ultrasonographic image of patient 3 showing an aneurismatic dilation at the splenic hilus and the relationship between the dilated splenic artery and aneurism.

**Figure 4:**
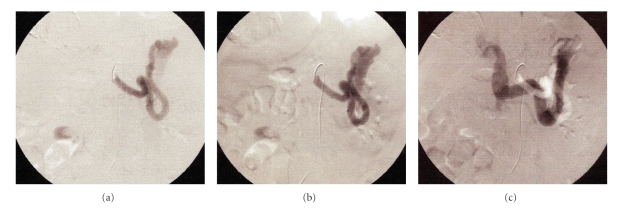
Angiographic images of patient 3 showing (a) dilated and tortuous splenic artery which ends in aneurism (b) similar to 4(a) with more filling of the aneurism and (c) dilated splenic vein together with splenic artery.

At her initial US, the liver was larger than normal with hypertrophic left lobe. The inner edges of the liver were minimally irregular. At right lobe, there was a calcified cyst with a diameter of 8 × 5 cm. Portal vein and splenic vein were dilated and tortuous. Hepatic veins and vena cava inferior were normal. The small bowel walls were thick and edematous. There was little ascites between the bowel loops in the pelvic region. Pancreas was normal. A large aneurism with irregular border at the hilus of the enlarged spleen was seen. This aneurism was on the splenic vein which has arterialized flow. Also dilated splenic artery was seen just below the aneurism. Her splenic artery was dilated from beginning just as originating from the celiac truncus and ends in the aneurism (Figure 3). Angiography confirmed US findings (Figure 4). Splenic artery was dilated from the beginning and ends in the aneurism. Dilated splenic vein filled early at the arterial phase.

Third grade esophageal varices and portal hypertensive gastropathy were seen at the upper gastrointestinal endoscopy. Small bowel follow through were normal other than minimal edematous mucosal plies. Her stool and blood tests for diarrhea were all normal. 

 Her abdominal pain and diarrhea continued together with deterioration of her general condition. At the control US it was seen that ascites increased up to the umbilicus level together with a discontinuation at the calcified hydatid cyst wall. So with the suspicion of hydatid cyst rupture an emergency operation was done. At operation, it was seen that the abdominal fluid looked likes simple ascites, without any debris in it. In spite of this a cystotomy and drainage operation was done to the calcified cyst at the liver. For the APF at the hilus of the spleen, the splenic artery only ligated from the proximal side of the fistula during the same urgent operation. But, at the postoperative period her general condition continued to deteriorate. At the control US a gross subcapsular splenic hematoma was detected. At the second operation, done 8 days later it was seen that the ligation of the splenic artery was inadequate and the APF still functioning. Splenectomy together with the end closure of the splenic artery was done. After the operation her condition stabilized. But she was lost 1 month later with sepsis and multiorgan failure.

## 3. Discussions

APF is an uncommon, but curable cause of portal hypertension. Portal hypertension disappears by closure of APF frequently. Ischemia of the liver parenchyma may develop with long-term APF, which decreases the perfusion pressure and steals highly oxygenated arterial blood flow [1]. Mostly, treatment of APF is recommended even in asymptomatic patients with high flow fistula to prevent life-threatening complications.

There are two treatment options for APF, surgery or interventional radiology. At the classic historical surgical treatment, excision of the fistula with direct repair of the vessels is the preferred method for extrahepatic APFs and partial hepatectomy and/or ligation of the hepatic artery for intrahepatic APFs. Interventional radiology is now considered as the treatment of first choice, because of the reduced cost and morbidity and repeated access availability. For transarterial embolization, a wide variety of materials can be used: N-butyl cyanoacrylate, gelfoam, steel coils, guide wire core, detachable balloons, and amplatzer occlusion device, and so forth [2, 5]. We suggest that every APF patient must be evaluated on a council including all of the related physicians and method of treatment is decided on an individual manner, according to the localization of the fistula, flow rate (low or high) of the fistula, existing liver reserve of the patient, simple-complex fistula, presence of collaterals or characteristics and number of feeding arteries.

Our first two patients were discussed on a council including gastroenterologists, gastroenterological surgeons, interventional radiologist and cardiovascular surgeons. For the first patient because of patient's age, closely normal liver function, unique and simple but high flow fistula without other extrahepatic portal anomalies, we decided on hepatic artery ligation. Also, it must be mentioned that, at that time interventional radiologic techniques suitable for high flow fistulas were not available for us. Surgical treatment result with an uneventful recovery and any short- or long-term complication did not occurr. For the second patient, because of his diminished liver function, presence of portal vein thrombosis and many extrahepatic collaterals within the abdomen angiographic treatment was preferred. While deciding the type of angiographic intervention, detachable balloon application was preferred instead of coil embolization because of the risk of coil migration. Although APF closure succeeded, his portal hypertension recurred 8 months later. Presence of portal vein thrombosis, probable liver damage related with long-term APF and other small connections (APFs) between the splanchnic arteries and veins was considered for the recurrence of portal hypertension. The third patient underwent an urgent operation (with suspicion of hydatid cyst rupture) before discussing and deciding about the type of the treatment. Also, splenic artery ligation done for the APF at this urgent operation was remained unsuccessful. With splenectomy and end closure of the splenic artery done at the second operation, the treatment of the fistula succeeded but the patient was lost due to complications related with operations.

Vauthey et al. [2] reported an aneurismatic dilatation, turbulent flow within aneurysm and reverse flow in portal vein as sonographic features in their APF cases. The pulsatile hepatofugal flow or arterial pattern in the portal vein frequently was demonstrated in APF patients [1, 4, 6–10]. Akpek et al. [11] and Gallego et al. [4] demonstrated that common hepatic artery and its branch which feeds aneurism had been dilated in addition to these. Mora et al. [12] and Lafortuna et al. [13] suggested APF when an echo-free focal hepatic lesion or dilated hepatic artery and portal vein had been demonstrated. But none of the papers had complete presentation of the sonographic features.

## 4. Conclusion

The diagnosis of all of our APF cases was by US. Besides the frequently reported features of APF, we detect that APF cases have six common sonographic features. We called these characteristics “sonographic pattern of APF”.

1. Both of the artery and the vein are seen as adjacent wide anechoic circles.
2. There is an aneurismatic dilation on the dilated vein. Other branches of the portal system are all normal.
3. The branch of artery communicating with aneurism is dilated along its all route just as originating from aorta (e.g., celiac truncus). All other arterial branches without communication with aneurism are completely normal.
4. There is a communication between artery and vein and this can be shown by US.
5. Doppler US shows a turbulent flow within aneurismatic dilatation. Also, arterial flow pattern is found in both of the vessels.
6. The flow of the vein is retrograde and arterialized.

US is a noninvasive and efficient diagnostic tool in APF. Also, it is useful in follow-up after treatment. Angiography is reserved to treatment or pretreatment evaluation. Although new diagnostic methods are developed, US is a very important widely used diagnostic tool. During routine common use of US, a person aware of this sonographic pattern can readily diagnose APF. In this way early diagnosis of APF can be done by US and give the curable treatment chance to the patient.
